# Preliminary Studies of the Immunomodulator Effect of the *Bougainvillea xbuttiana* Extract in a Mouse Model

**DOI:** 10.1155/2015/479412

**Published:** 2015-03-16

**Authors:** Lluvia Arteaga Figueroa, Leticia Barbosa Navarro, Martin Patiño Vera, Vera L. Petricevich

**Affiliations:** ^1^Facultad de Medicina, Universidad Autónoma del Estado de Morelos (UAEM), Laboratorio de Inflamación y Toxicología, Calle Iztaccihuatl Esquina Leñeros, Colonia Volcanes, 62350 Cuernavaca, MOR, Mexico; ^2^Instituto de Biotecnología de la Universidad Nacional Autónoma de México, Mexico

## Abstract

*Bougainvillea xbuttiana* is used as an analgesic in folk medicine in Mexico. The purpose of the present study was to determine the effects of the ethanolic extract from *B. xbuttiana* on macrophages activities. The phytochemical screening was performed for determine the presence of alkaloids, flavonoids, triterpenes, and saponins. The effects of *B. xbuttiana* were analyzed using the macrophages activities as determined by the H_2_O_2_ release, spreading and phagocytic index, vacuoles formation percentage, and mediators production. The viability percentage was determined in live cells after fixing and staining with crystal violet. The presence of H_2_O_2_ in macrophages was performed by using the peroxidase-phenol red solution. The cytokine production was determined by two assays, ELISA for detection of IL-6, IL-10, and IFN-*γ* and biological assay for TNF detection. The results showed that the *Bxb* extract dose-dependent manner produces (a) an increase in levels of H_2_O_2_ and spreading and vacuoles formation percentages, (b) a decrease in phagocytic index and in the amounts of TNF, IL-6, and IFN-*γ*, and (c) an increase significant in IL-10 and NO production. This study indicates that the ethanolic extract from *Bougainvillea xbuttiana* was able to activate macrophages. The combination of these results suggests that this extract has an immunomodulator effect.

## 1. Introduction

There are a lot of archaeological evidences that humans used medicinal plants for better living, reducing disease, and improving quality of life. Several studies have described the use of plants in traditional medicine for many years and have recently gained much importance in the field of pharmacological industries. The knowledge and assessments of the biological properties of extracts from plants can serve as a source of future drug candidates in many areas of health [1].

The relevant literature shows that many plants or plant products have been used as an alternative source for the treatment of different diseases. Several of these herbs have been used to exert immunomodulatory effect in the treatment of various diseases [2]. Medicinal plants with immunomodulatory activity are used for cases of organ transplant rejection or for treatment of the various autoimmune diseases [3, 4].

Immunomodulation is a process used to describe an increase or decrease in the magnitude of immune response. The immunostimulants are those compounds that act in an enhancement of immunes reactions and are involved in nonspecific system stimulation [5]. In contrast, the immunosuppressive compounds are those capable of decreasing the resistance against infections and stress caused by environmental factors or chemotherapy [6].

Medicinal plants can inhibit or stimulate immune response which could be useful in the treatment of various human diseases. In particular, medicinal plants capable of inhibiting the cellular and humoral responses could have useful applications in the treatment of immunological disorders [7].

The state of macrophages activation and T and B lymphocytes plays a major role in the pathogenesis of immune-mediated disorders [8]. Macrophages are essential members of the innate immune system and, together with neutrophils, eosinophils and natural killer cells are a first line of defense to identify, eliminate or contain invading microorganisms and toxic macromolecules. Macrophages are pivotal key in the maintenance of tissue homeostasis and are responsible for detecting, engulfing, and destroying pathogens [9]. In response to injury, macrophages bind to invading pathogens and deliver them to other components of adaptive immunity, which is constituted by antibody and cell mediated responses that are performed by different lymphocyte cells, B cells, and T cells, respectively [9–11]. During macrophage activation, several compounds are released such as cytokines, reactive oxygen species, nitric oxide, and lipid inflammatory mediators which are implicated in the inflammatory response [12]. With respect to cytokines, they regulate the intensity and duration of the immune response by stimulating or inhibiting the activation, proliferation, and differentiation of various cells and by regulating their secretion of antibodies or other cytokines [12–14]. Inflammatory cytokines can be classified into two groups: those involved in acute inflammation, named proinflammatory cytokines, and those responsible for reducing inflammation, named anti-inflammatory cytokines [15–17]. The proinflammatory cytokines are cytokines which promote systemic inflammation and include interleukin-1 (IL-1), interferon-gamma (IFN-*γ*), and tumor necrosis factor (TNF). By contrast, anti-inflammatory cytokines are those responsible for regulating and maintaining homeostasis. Major anti-inflammatory cytokines include IL-4, IL-6, IL-10, and IL-13 [17–19].

Nitric oxide (NO) plays an important role in various functions including vasoregulation, neurotransmission, regulation of immune responses and cytotoxic activity against tumor cells and a variety of pathogens by macrophages mid. It is involved in the pathogenesis and control of infectious diseases [20]. NO is derived from L-arginine in a reaction catalyzed by nitric oxide synthase (NOS). The NOS has different isoforms: the constitutive NOS (cNOS) and inducible NOS (iNOS) [21, 22]. Endotoxins or cytokines induce the expression of NOS that is capable of generating an amount of NO greater than the other types of NOS [21, 22]. The increment of NO production contributes to tissue damage inflammatory and infectious diseases including septic shock and stroke [21–23].

For a long time, the compounds derived from medicinal plants were the basis for use as drugs. Various compounds of plants have been described for their anti-inflammatory or immunomodulator activity.

*Bougainvillea* is from family Nyctaginaceae which has 30 genera distributed mainly in tropical climates. It is reported in literature the existence of 18 different species. In Cuernavaca, Morelos and other states of Mexico the predominant species is the* B. xbuttiana*.* Bougainvillea* is widely used as a medicinal plant in the states of central and southern Mexican territory. Infusions prepared from flowers or leaves are used for gastrointestinal and respiratory disorders. With respect to gastrointestinal disorders, the infusion is used as antidiarrheal and to reduce the acid content of the stomach. In the case of respiratory disorders, the infusion is used to treat cough, asthma, bronchitis, and whooping cough. For the treatment of these diseases, the infusions are prepared from flowers or leaves, which are administered orally. For other parts of the plant, root decoction is used for fevers and as a laxative [24, 25]. For the many attributes found by the use of* Bougainvillea*, we decided to examine its effects on macrophage activation, which was determined by assays phagocytic index, vacuoles formation, peroxide hydrogen, cells expansion, and mediators production.

## 2. Materials and Methods

### 2.1. Chemicals, Reagents, and Buffers

Fetal calf serum (FCS) and RPMI-1640 medium were purchased from Sigma (St. Louis, MO, USA). Capture and detection antibodies and recombinant cytokines were purchased from BD Biosciences Pharmingen (USA), and recombinant TNF was purchased from Boehringer Mannheim (Mannheim, Germany).

### 2.2. Plant Material and Extraction of* Bougainvillea xbuttiana*

The plant specimens were collected from Cuernavaca (Morelos, Mexico).* Bougainvillea xbuttiana* was identified from a voucher specimen (23683) in Herbarium HUMO, CIByC-UAEM. Extraction method is the crucial first step in the analysis of medicinal plants, because it is necessary to extract the desired chemical components from the plant materials for further characterization. Patent MX/a/2011/813522 provides details in extraction method of* Bougainvillea xbuttiana* flowers named as* Bxb* [25].

### 2.3. Phytochemical Tests

Phytochemicals screening methods for detecting the presence of secondary metabolites in* Bxb* were (a) Dragendorff test for detecting alkaloids; (b) Shinoda test [26] for detecting flavonoids; (c) the foam test [26, 27] for determining saponins; (d) Liebermann-Buchard test for detecting triterpenes; (e) Folin-Ciocalteau Method to quantify total phenols contents [28] and (f) reaction with AlCl_3_ in base medium for quantifying total flavonois contents [28].

### 2.4. Animals

Female mice of CD1 strain weighing 20–25 g were purchased from Facultad de Medicina, UAEM. The animals were kept under strict ethical conditions according to international guidelines for the care and use of laboratory animals. The experiments designed for this study were approved by the Committee of Experimental Animal Administration of the university with protocol number 13MM/2011, [29].

### 2.5. Peritoneal Macrophages

Female mice of the CD1 strain were sacrificed and peritoneal cells were collected by peritoneal lavage as previously described by Cohn and Benson, 1965 [30]. The obtained cell suspension was centrifuged and the cells were resuspended in RPMI-1640 supplemented with 10% FCS and adjusted at a final concentration of 1 × 10^6^ cells/mL and distributed in 96-well microplates. Cell cultures were incubated for 2 hours at 37°C with an atmosphere of 5% CO_2_. Then nonadherent cells were removed and adherent cells of different concentrations of the* Bxb* were added. At different times of incubation, the supernatants were collected and frozen at −20°C until assessment of mediators.

### 2.6. Viability Assay

Culture supernatants from macrophages treated or not treated with* Bxb* incubated for 24, 48, 72, and 96 hours were discarded and the live cells were fixed and stained with crystal violet for 10 minutes at room temperature. Excess stain was removed through washing and absorbance was determined in a microplate reader with a filter of 620 nm. To calculate the viability percentage, the following formula was used: [(*A*_sample_/*A*_control_) × 100] [31].

### 2.7. Phagocytosis Assays

The probe to determine the phagocytic index was performed as described by Zebedee et al., 1994 [32]. Briefly, cultures of macrophages at a concentration of 1 × 10^6^ cells/mL treated or not treated with* Bxb* were incubated at 37°C with an atmosphere of 5% CO_2_. At the end of 24, 48, 72, and 96 hours of incubation, the supernatants were collected and a solution consisting 1 : 5 of opsonized yeast was added. After 1 hour of incubation, the supernatants were removed and safranin solution was added for 40 seconds. The cells were examined by using optical microscope at 40x. The phagocytic index (PI) was determined as follows: number of macrophages with internalized yeast/100.

### 2.8. Vacuole Formation Assay

Macrophage cultures were incubated with RPMI-1640 medium plus 5% FCS and 1 mM NH_4_Cl/mL and then were treated with 2.9, 29, and 290 *μ*g/mL* Bxb* at 37°C with 5% CO_2_ [33]. After 24, 48, 72, and 96 hours of incubation, cells were stained by 0.05% neutral red for 5 minutes. Cells were then washed with PBS containing 0.2% BSA, 70% ethanol, and 0.37% HCl. The absorbance was determined using the microtiter plate reader with a filter of 540 nm. Vacuole formation percentage was calculated by the following formula: [(*A*_sample_ − *A*_control_/*A*_control_) × 100].

### 2.9. Spreading Index

Spreading index was determined by the method described by Arruda et al. (2004) [34]. In brief, the suspension of 1 × 10^6^/mL macrophages was distributed in microplate which contained slides to assess cell adhesion. Cultures treated or not treated with 2.9, 29, and 290 *μ*g/mL* Bxb* were incubated at 37°C with 5% CO_2_. After 24, 48, 72, and 96 hours of incubation, the slides were removed and immersed in methanol and then stained with crystal violet. After drying, the cells were observed with an optical microscope of 40x magnification. The spreading index (SI) corresponds to a percentage value of 100 macrophages.

### 2.10. Hydrogen Peroxide

Hydrogen peroxide production in macrophage was determined as described by Pick and Mizel, in 1981 [35]. Macrophage cultures treated or not treated with 2.9, 29, and 290 *μ*g/mL* Bxb* were incubated at 37°C with 5% CO_2_. After 24, 48, 72, and 96 hours of incubation, the supernatants were removed. Directly on the cells was added a solution of phenol with 140 mM NaCl; 10 mM K_2_PO_4_; 5.5 mM dextrose; and 5.5 mM horseradish peroxidase. Absorbance was determined in the microplate reader with 620 nm filter and the hydrogen peroxide concentration was determined by comparison with a standard curve.

### 2.11. Cytokines ELISA and Biological Assays

#### 2.11.1. ELISA Assay

Cytokines such as IL-6, TNF, and IFN-*γ* present in the supernatant of macrophages from CD1 mice treated or not treated with* Bxb* were detected by ELISA assay [36]. All assays were performed according to manufacturer's instructions. Minimal detection for IL-6 and IL-10 was 10 pg/mL and for IFN-*γ* 100 pg/mL.

#### 2.11.2. Biological Assay

It was used to measure TNF present in the supernatant of macrophages treated or untreated with* Bxb* [37]. The percentage of TNF cytotoxicity on L929 cells was calculated by the following formula: (*A*_control_ − *A*_sample_/*A*_control_) × 100. TNF amounts are expressed as pg/mL estimated from the ratio of a 50% cytotoxic dose of the test to that of standard mouse recombinant TNF.

### 2.12. Nitrite Determination

Nitrite in the presence of macrophage supernatants of treated and untreated mice with* Bxb* was determined as previously described by Keller et al., 1990 [38]. Briefly, macrophages supernatants from treated or untreated* Bxb* for different times were mixed with freshly prepared Griess reagent (0.1% naphthalenediamine hydrochloride, sulphonylamide 1%, and 3% H_3_PO_4_). Absorbance was measured in a microplate reader with 540 nm filter. The amount of nitrite was determined by a standard curve of NaNO_2_. Minimal detection for nitrite was 1.25 nM.

### 2.13. Statistical Analyses

The data were statistically analyzed by Student's *t*-test. A value of *P* < 0.01 was considered significant.

## 3. Results

### 3.1. Qualitative and Quantitative Measurements

The standardized extracts were as follows: one is based on identifying and the second based on quantifying a* Bxb* to a characteristic chemical. The phytochemical test was positive for secondary metabolites such as alkaloids, triterpenes, flavonoids, and saponins (Table 1). We have previously reported the quantitative identification of phytochemical compounds in* Bxb* extract [28].

### 3.2. Effects of* Bxb* on Macrophages Viability

**Figure 1:**
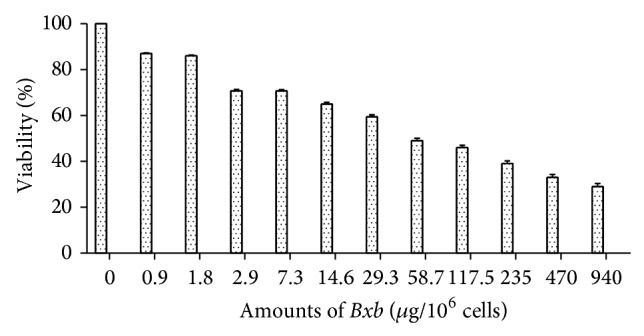
Effect of* Bxb* on peritoneal macrophage viability. Peritoneal macrophages were obtained and treated* in vitro* with different concentrations of* Bxb* as described above. The viability percentage was observed at 48 h after extract exposition. Each bar represents the mean value of samples from three experiments in different groups of five mice. Statistical differences between the treatments were *P* < 0.05.

To determine the effect of* Bxb extract* on viability of peritoneal macrophages, these cells were obtained by peritoneal lavage, cultured and treated* in vitro* with different concentrations of* Bxb*, and incubated for distinct time. The effect of* Bxb* was determined by measuring viability percentage. The* Bxb* induced dose-dependent inhibition on cell viability (Figure 1). Peritoneal macrophages treated during 48 hours with 2.9 up to 14.6 *μ*g/mL of* Bxb* showed viability percentage decreased by 30% (Figure 1). For groups of cells exposed to 29.3 up to 237 *μ*g/mL, the viability decreased by 51% up to 60%. The lowest viability percentage was obtained in macrophages treated with 470 and 940 *μ*g/mL of* Bxb* (Figure 1). We also determined that a concentration of* Bxb* extract that induces the decreasing in 50% macrophage viability was 29 *μ*g/mL. After determining the optimal interaction between cell and* Bxb*, we designed the further analysis using the concentrations as follows: 0, 2.9, 29, and 290 *μ*g/mL.

**Figure 2:**
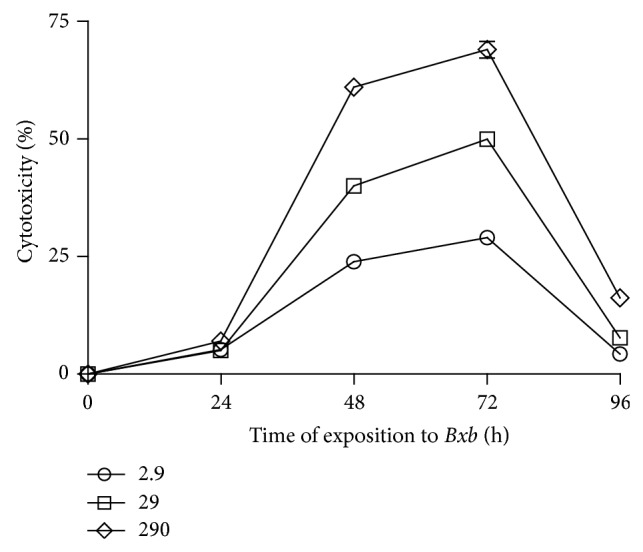
Cytotoxic activity of* Bxb*. Peritoneal macrophages were obtained and exposed* in vitro* to 2.9, 29, and 290 *μ*g/mL of* Bxb* as described above. The cytotoxic percentage was observed at different times after* Bxb* exposition. Each point represents the mean value of samples from four experiments in different groups of five mice. Statistical differences between the treatments were *P* < 0.05.

The kinetics of cytotoxicity percentage of peritoneal macrophages treated or not treated with different concentrations of* Bxb* during distinct time periods is shown in Figure 2. In cultures of macrophages treated with 2.9 *μ*g/mL of* Bxb* the cytotoxicity percentage was 5%, 24%, and 29% for 24, 48, and 72 hours, respectively (Figure 2). The cytotoxicity percentage in macrophages treated with 29 *μ*g/mL of* Bxb* was 5%, 40%, and 50% for 24, 48, and 72 hours, respectively (Figure 2). In macrophages treated with 290 *μ*g/mL of* Bxb*, the cytotoxicity percentages were 7%, 61%, and 69% for 24, 48, and 72 hours, respectively (Figure 2).

### 3.3. Effects of* Bxb* on Macrophages Activities

To evaluate the activities of macrophages, the cells were obtained by peritoneal lavage, cultivated and treated* in vitro* with different concentrations of* Bxb*, and incubated for distinct time. The effects of* Bxb* on macrophages were performed by measuring H_2_O_2_, phagocytic index, spreading index, and percentage of vacuoles formation.

**Figure 3:**
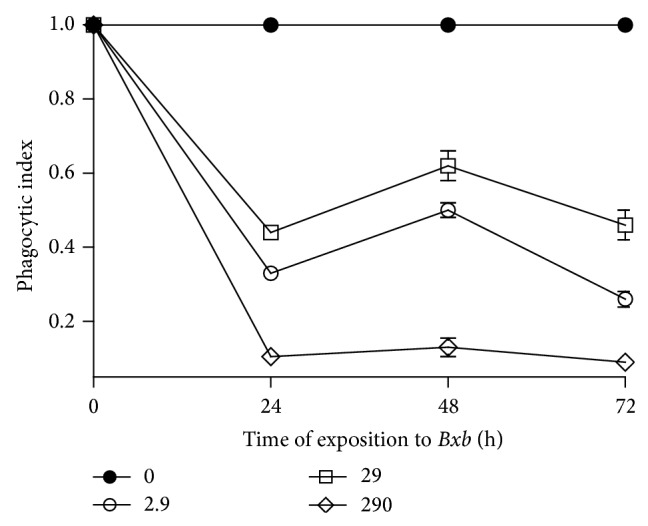
Phagocytosis index. Peritoneal macrophages were obtained and exposed to 2.9, 29, and 290 *μ*g/mL of* Bxb* and the phagocytic index was determined as described above. Each point represents the mean value of samples from four experiments in different groups of five mice. Statistical differences between the treatments were *P* > 0.05.

As shown in Figure 3, the macrophage groups treated with 2.9, 29 and 290 *μ*g/mL of* Bxb* during different periods of time the phagocytic index was significantly lower than those obtained from untreated macrophages cultures (*P* < 0.001).

**Figure 4:**
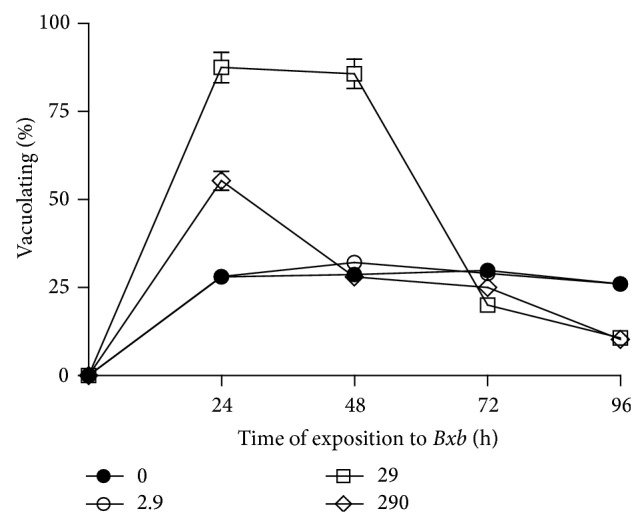
Vacuole formation. Peritoneal macrophages were obtained and exposed to 2.9, 29, and 290 *μ*g/mL of* Bxb* as described above. After different times of incubation at 37°C in an atmosphere of 5% CO_2_, the cells were stained with neutral red for 5 min. The absorbance was determined at 540 nm, and the results were expressed as described above. Each point represents the mean value of samples from four experiments in different groups of four mice. Statistical differences between the treatments were *P* < 0.05.

The vacuoles formation was quantified by a period of 24 up to 96 hours, using the neutral red assay. The vacuolating percentage in macrophages treated with 2.9 *μ*g/mL of* Bxb* was similar when compared with untreated macrophages (Figure 4). In contrast, when the macrophages were treated with 29 *μ*g/mL of* Bxb* extract during 24 and 48 hours, the vacuolating percentage was significantly higher when compared with the results obtained in control macrophages (*P* < 0.001), decaying thereafter. In case of macrophages treated with 290 *μ*g/mL of* Bxb* for 24 hours, the vacuolating percentage was significantly higher than those obtained in untreated macrophages (*P* < 0.001), decaying thereafter.

**Figure 5:**
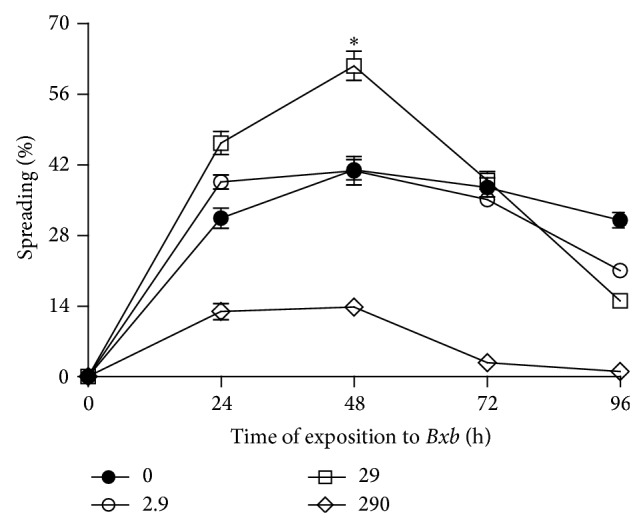
Spreading index. Peritoneal macrophages were obtained and exposed to 2.9, 29, and 290 *μ*g/mL of* Bxb* and the spreading percentage was determined as described above. Each point represents the mean value of samples from four experiments in different groups of five mice. Statistical differences between the treatments were *P* < 0.05.

In Figure 5 is shown the spread index. In macrophages treated with 2.9 *μ*g/mL of* Bxb*, the percentage of spreading was similar to that obtained from untreated macrophages. For groups of macrophages treated with 29 *μ*g/mL of* Bxb* during 48 hours, the percentage of spreading presented was significantly higher when compared with those results observed in untreated macrophages (*P* < 0.001). In contrast, in macrophages treated with 290 *μ*g/mL of* Bxb*, the spreading percentages were significantly lower than those observed for the untreated macrophages (*P* < 0.001).

**Figure 6:**
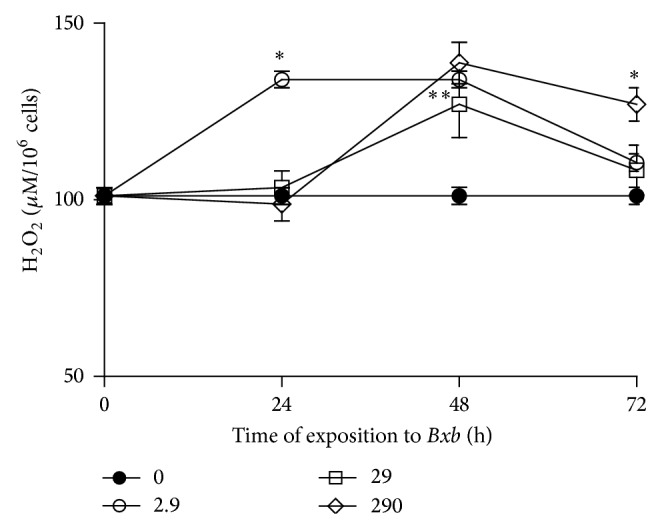
Hydrogen peroxide production. Peritoneal macrophages were obtained and exposed to 2.9, 29, and 290 *μ*g/mL of* Bxb* and the hydrogen peroxide production was determined directly on cells as described above. Each point represents the mean value of samples from four experiments in different groups of five mice. Statistical differences between the treatments were *P* < 0.05.

The levels of hydrogen peroxide present in supernatants of macrophages treated or not treated with* Bxb* are shown in Figure 6. The highest levels of H_2_O_2_ were observed among peritoneal macrophages treated with 2.9 *μ*g/mL of* Bxb* for 24 and 48 hours. For groups of macrophages treated with 29 and 290 *μ*g/mL of* Bxb* during 48 hours, the levels of H_2_O_2_ were significantly higher than those obtained in untreated macrophages (*P* < 0.001).

### 3.4. Effect of* Bxb* on Cytokines and NO Production

To determine the capacity of* Bxb* to stimulate the production of cytokines and NO, groups of mice were sacrificed and their macrophages were collected by peritoneal lavage and they were* in vitro *treated with different concentrations of* Bxb* for distinct time periods.

**Figure 7:**
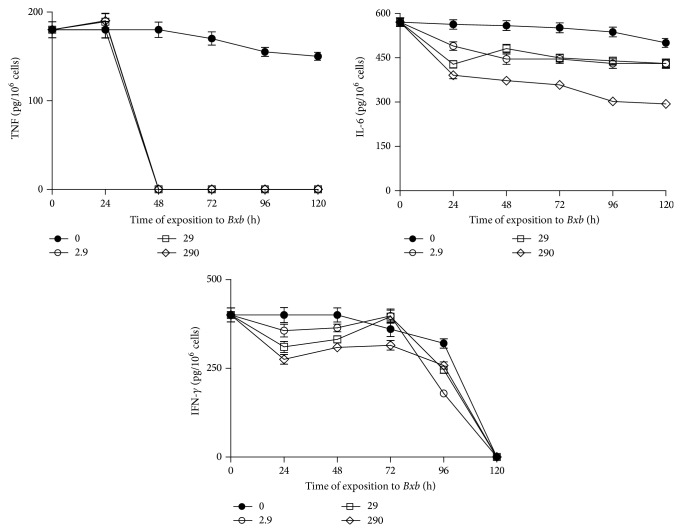
Proinflammatory cytokines production. Peritoneal macrophages were obtained and exposed* in vitro* to 2.9, 29, and 290 *μ*g/mL of* Bxb* as described above. At different times, the levels of IL-6 and IFN-*γ* were determined by ELISA assay using monoclonal antibodies as the probe. The levels of TNF were determined by standard assay with L929 cells. Each point represents the mean value of samples from four experiments in different groups of five mice. Statistical differences between the treatments were *P* < 0.05.

The kinetics of proinflammatory cytokines production is shown in Figure 7. TNF production was significantly lower in macrophages treated with 2.9, 29, and 290 *μ*g/mL of* Bxb* for all time periods studied here than that obtained in untreated macrophages (*P* < 0.001). Treatment of macrophages with 2.9, 29 and 290 *μ*g/mL of* Bxb* caused significant decrease in the production of IL-6 when compared with untreated macrophages (*P* < 0.01). Figure 7 also shows that the levels of IFN-*γ*, from macrophages treated with 2.9, 29, and 290 *μ*g/mL of* Bxb*, were significantly decreased when compared with those levels obtained from untreated macrophages cultures (*P* < 0.01).

**Figure 8:**
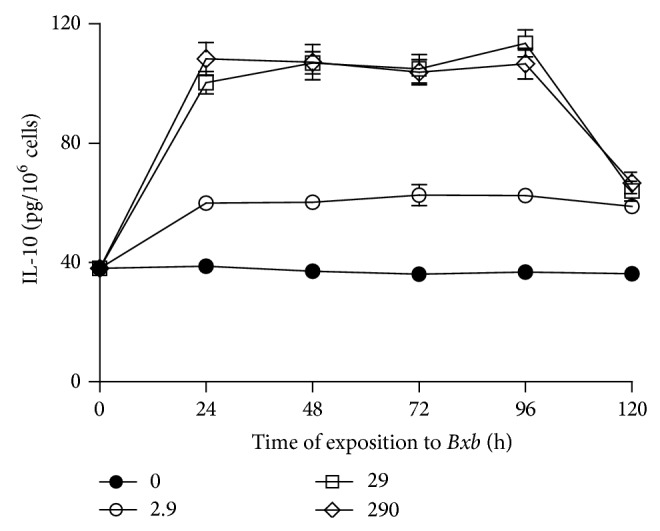
Anti-inflammatory cytokines production. Peritoneal macrophages were obtained and exposed to 2.9, 29, and 290 *μ*g/mL of* Bxb* and at different times the levels of IL-10 were assayed by ELISA assay using monoclonal antibodies as the probe. Each point represents the mean value of samples from four experiments in different groups of five mice. Statistical differences between the treatments were *P* < 0.05.

In Figure 8 is shown the kinetics of anti-inflammatory cytokines production. The* in vitro* treatment of macrophages with* Bxb* resulted in IL-10 production. For macrophages cultures treated with 2.9, 29, and 290 *μ*g/mL of* Bxb*, the IL-10 levels were significantly higher than those obtained from untreated macrophages cultures (*P* < 0.01).

**Figure 9:**
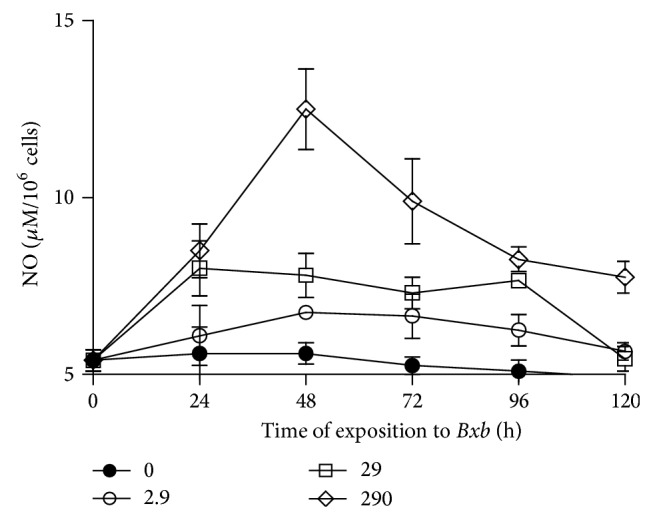
NO production. Peritoneal macrophages were obtained and exposed to 2.9, 29, and 290 *μ*g/mL of* Bxb* and at different times the levels of NO were determined by Griess colorimetric reaction. Each point represents the mean value of samples from four experiments in different groups of five mice. Statistical differences between the treatments were *P* < 0.05.

As shown in Figure 9, the* Bxb* increased the NO production in macrophages in a concentration-dependent manner. For macrophages cultures treated with 2.9, 29, and 290 *μ*g/mL of* Bxb*, the NO production was significantly higher than those obtained from untreated macrophages (*P* < 0.01), decaying thereafter.

## 4. Discussion

Many natural products have played a significant role in maintaining and improving the quality of human life. The modulation of immune response to alleviate many diseases has been one mechanism of interest for pharmacology studies. A great number of medicinal plants have been shown to stimulate or inhibit immune response. In the present study, ethanolic extract obtained from* Bxb* native from Mexico was investigated for its effects in macrophage activities and mediators production. For this purpose, we firstly evaluated the cytotoxic activity of the* Bxb* extract in peritoneal macrophages. The* Bxb* showed cytotoxic activity dose-dependent manner.

The phytochemical screening of secondary metabolites in* Bxb* revealed the presence of alkaloids, flavonoids, triterpenes, and saponins. These compounds have been shown to contain molecules with potent effects on the host immune system by either stimulant or suppressor of immune response.

Exposure of organisms to bacterial infection can result in the activation of defense mechanisms which include phagocytosis, cell motility, and spreading. In the present study, it has been clearly demonstrated that* Bxb* exerts inhibitory effect on the phagocytic ability by macrophages* in vitro* culture. Similar results have been reported with extracts from other plant families [39]. Particles internalization by macrophages results in the generation of vacuoles. In this study, we also observed that peritoneal macrophages exposed to 29 *μ*g/mL of* Bxb* presented high presence of vacuoles. In these cells, the plasma membrane was well-preserved which is characteristic of cell viability. The pronounced spreading has been considered a marker of macrophages and is a typical morphological characteristic of activated macrophages [39, 40]. In this study, we observed that 29 *μ*g/mL of* Bxb* extract was capable to induce the spreading in macrophages. The spreading provides morphological evidence of considerable cellular modifications in cytoskeleton reorganization and changes of membrane protein activities and expression [41]. Therefore, the phagocytosis index observed in* Bxb* treatment of macrophages was coordinated with macrophage activity. These parameters thus indicate that* Bxb* has an efficient capacity to activate macrophages. Hydrogen peroxide is important in cell signaling and it is an effector molecule for microbicidal and cytotoxic response of macrophages after stimulation and the development of inflammation. In our study, it was also found that* Bxb *induces the hydrogen peroxide production in peritoneal macrophages. Taken together, these data demonstrated that the* Bxb* is able to generate the macrophage activation.

Macrophages activated produce and release products including several cytokines, inorganic reactive radicals, reactive oxygen intermediates, and reactive nitrogen intermediates with biological activities [9, 10]. In this study, we exposed peritoneal macrophages to* Bxb* to demonstrate the production of cytokines. The proinflammatory cytokines are essential for initiating the inflammation process leading to tissue destruction. These cytokines induce tissue destruction and reduce the capacity to repair damaged tissue by stimulating the production of other mediators [42]. Among the proinflammatory cytokines, IL-6 is one of most important mediators of fever and acute-phase response. TNF has cytostatic and cytocidal effects and the ability to cause apoptosis [43]. The production of IFN-*γ* and IL-10 provides important protection to cells and tissues against deleterious effects of free radicals [44]. The reduction of these cytokines may reflect the anti-inflammatory activity of plant extracts. Various studies have shown the immunomodulatory effects from plants extracts. In our study, it was found that the* Bxb* was capable of decreasing the proinflammatory cytokines such as TNF, IL-6, and IFN-*γ* production in macrophages and promoted the production of anti-inflammatory cytokines, such as IL-10. These combined results suggest an immunomodulatory activity of the extract* Bxb*. The reduction in the biological activity of IL-1 or TNF is accomplished by highly specific strategies for the treatment of patients with inflammatory diseases [9, 10].

Nitric oxide is a mediator related to cell activation which contributes to the death or inhibition of a variety of pathogens [20, 45]. In our study, it was found that 29 *μ*g/mL of* Bxb* was able to induce the NO production. These results are in agreement with other studies that have demonstrated that NO has an important regulatory role in the various types of inflammatory processes. Nitric oxide is synthesized in large quantities by activated macrophages and has been demonstrated to be involved in the pathogenesis of acute and chronic inflammatory conditions.

In conclusion, the* B. xbuttiana* extract is able to suppress the production of mediators, such as TNF, IFN-*γ*, and IL-6, and enhanced the production of NO and IL-10 in a dose-dependent manner. Further detailed studies are required to identify the active constituents and their mechanism for this effect.

## Figures and Tables

**Table 1:** Qualitative identification of phytochemical compounds in *Bxb*.

Alkaloids	+++
Flavonoids	+++
Saponins	+
Triterpenes	++

Absent (−), present (+), moderate (++), and abundant (+++).
